# Survival and cause‐specific mortality of harvested willow ptarmigan (*Lagopus lagopus*) in central Norway

**DOI:** 10.1002/ece3.6754

**Published:** 2020-09-15

**Authors:** Markus F. Israelsen, Lasse F. Eriksen, Pål F. Moa, Bjørn R. Hagen, Erlend B. Nilsen

**Affiliations:** ^1^ Terrestrial Division Norwegian Institute for Nature Research (NINA) Trondheim Norway; ^2^ Faculty of Biosciences and Aquaculture Nord University Steinkjer Norway; ^3^ Department of Biology Centre for Biodiversity Dynamics Norwegian University of Science and Technology Trondheim Norway

**Keywords:** cause‐specific mortality, demography, grouse, harvest, survival

## Abstract

Survival is a key demographic component that often varies as a result of human activities such as recreational harvest. Detailed understanding of seasonal variation in mortality patterns and the role of various risk factors is thus crucial for understanding the link between environmental variation and wildlife population dynamics and to design sustainable harvest management systems. Here, we report from a detailed seasonal and cause‐specific decomposition of mortality risks in willow ptarmigan (*Lagopus lagopus*) in central Norway. The analyses are based on radio‐collared (*n* = 188) birds that were monitored across all seasons, and we used time‐to‐event models for competing risks to estimate mortality patterns. Overall, annual survival was estimated at 0.43 (SE: 0.04), with no distinct difference among years (2015/16 to 2018/19) or between sexes. Analysis of mortality risk factors revealed that on the annual basis, the risk of harvest mortality was lower than the risk of dying from natural causes. However, during the autumn harvest season (September–November), survival was low and the dominating cause of mortality was harvest. During winter (December–March) and spring seasons (April–May), survival was in general high and did not vary between males and females. However, during the spring season, juveniles (i.e., birds born last year) of both sexes had lower survival than adults, potentially because they are more prone to predation. During the summer season (June–August), females experienced a higher hazard than males, underlining the greater parental investment of females during egg production, incubation, and chick rearing compared to males. Our analyses provide unique insight into demographic and seasonal patterns in willow ptarmigan mortality risks in a harvested population and revealed a complex interplay across seasons, risk factors, and demographic classes. Such insight is valuable when designing sustainable management plans in a world undergoing massive environmental perturbations.

## Graphical abstract

We studied willow ptarmigan mortality patterns in a managed population. Our analyses provide unique insight into demographic and seasonal patterns in willow ptarmigan mortality risks in a harvested population and revealed a complex interplay across seasons, risk factors, and demographic classes.

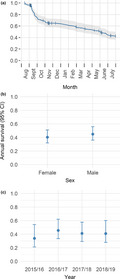

## 1 INTRODUCTION

Population dynamics are driven by temporal and spatial fluctuations in demographic rates that together determine the population growth rate *λ* (Sæther & Bakke, 2000; Sæther, Ringsby, Bakke, & Solberg, 1999). Both survival and reproductive output contribute to the observed variation, and their general contribution varies both in time and in space (Nilsen et al., 2009; Sæther & Bakke, 2000). In addition, research focusing on the evolution of life history strategies has found that species can be classified along a slow–fast continuum (Bielby et al., 2007; Sæther & Bakke, 2000; Stearns, 1983). Generally, fast‐living species have low survival and high reproductive output, whereas slow‐living species have high survival rates and lower reproductive output (Sæther et al., 2013). Species on opposite ends of the continuum also differ in the way age‐specific survival contributes to the population growth rate (Sæther et al., 2013). The potential contribution of adult survival is higher in slow‐living species (Sæther & Bakke, 2000), whereas the potential contribution of early life survival is higher in fast‐living species (Bielby et al., 2007). Annual mortality patterns are often very different for species on different ends of the continuum. Therefore, understanding the spatiotemporal variation in survival and cause‐specific mortality rates is imperative for understanding the population dynamics of wildlife species (DelGiudice, Riggs, Joly, & Pan, 2002; Heisey & Patterson, 2006; Murray, 2006).

Previous studies have reported that demographic factors such as sex and age can significantly affect the survival probability (Beauplet, Barbraud, Dabin, Küssener, & Guinet, 2006; Caizergues & Ellison, 1997; Shackell, Shelton, Hoenig, & Carscadden, 1994) and mortality causes (Asmyhr, Willebrand, & Hörnell‐Willebrand, 2012; Chilvers & MacKenzie, 2010; Delgiudice, Fieberg, Riggs, Powell, & Pan, 2006; Hannon, Gruys, & Schieck, 2003) of a range of avian species. Moreover, in temporally variable environments mortality risk might vary through time (Crespin et al., 2002; Gauthier, Pradel, Menu, & Lebreton, 2001), and the ability to deal with unpredictable environmental conditions may vary between life stages (Delgiudice et al., 2006). For instance, adult survival is often reported to be higher and less variable than juvenile survival (Guillemain et al., 2013). Finally, certain seasons within the year may also place more stress on one sex than the other due to variation in the sex‐specific costs of reproduction, such as the energy‐demanding process of egg production (Nilsson & Råberg, 2001) and incubation (Haftorn & Reinertsen, 1985) for female birds or risky behavior undertaken by males in the mating season (Hannon et al., 2003).

In wild vertebrate populations, individuals are typically facing competing risks from a range of different sources, and these sources might have different intensities in different times of the year. In exploited populations, previous studies have demonstrated that harvest‐related mortality risks may be close to or even higher than natural mortality risks in parts of the year (Rolland, Hostetler, Hines, Percival, & Oli, 2010; Sandercock, Nilsen, Brøseth, & Pedersen, 2011). Harvest mortality is often assumed to be partially compensated through reduced natural mortality (Pedersen et al., 2004). However, this may only be true at low harvest rates, where harvest mortality above certain levels may be increasingly additive or even superadditive (Sandercock et al., 2011). Knowledge of such thresholds and any compensatory mechanisms is thus essential information for sustainable harvest management (Brøseth, Tufto, Pedersen, Steen, & Kastdalen, 2005). For harvested wildlife populations, understanding the interplay between harvest‐induced mortality and other natural mortality sources is important in order to establish sustainable harvest strategies (Sandercock et al., 2011).

Willow ptarmigan (*Lagopus lagopus* L.) is a valued game species and is hunted in many parts of its distributional range (Storch, 2007), including Scandinavia (Aanes, Engen, Sæther, Willebrand, & Marcström, 2002; Asmyhr et al., 2012). After a strong decline in population numbers, the willow ptarmigan was in 2015 classified as near threatened (NT) in the Norwegian Red List for Species (Henriksen & Hilmo, 2015), fueling a debate of harvest effects on population development (Breisjøberget, Odden, Storaas, Nilsen, & Kvasnes, 2018). This makes the Norwegian willow ptarmigan population a highly relevant case study for a detailed examination of variation in mortality patterns for a managed wildlife species. To this end, we used 5 years of telemetry data from central Norway to characterize annual and seasonal mortality risks for different sex‐ and age classes. In particular, we first (a) estimated annual survival rates for the different demographic groups in the population. Second, (b) we decomposed the annual cycle into distinct seasons and assessed sex‐ and age‐ effects within seasons. Finally, (c) we estimated the relative natural and harvest‐induced risks using a competing risk formulation, and estimated seasonal patterns in hazard rates. In sum, these analyses will provide an important description of how different hazards shape the annual mortality patterns for different demographic groups in a wildlife population.

## 2 MATERIALS AND METHODS

### 2.1 Study area

**FIGURE 1:**
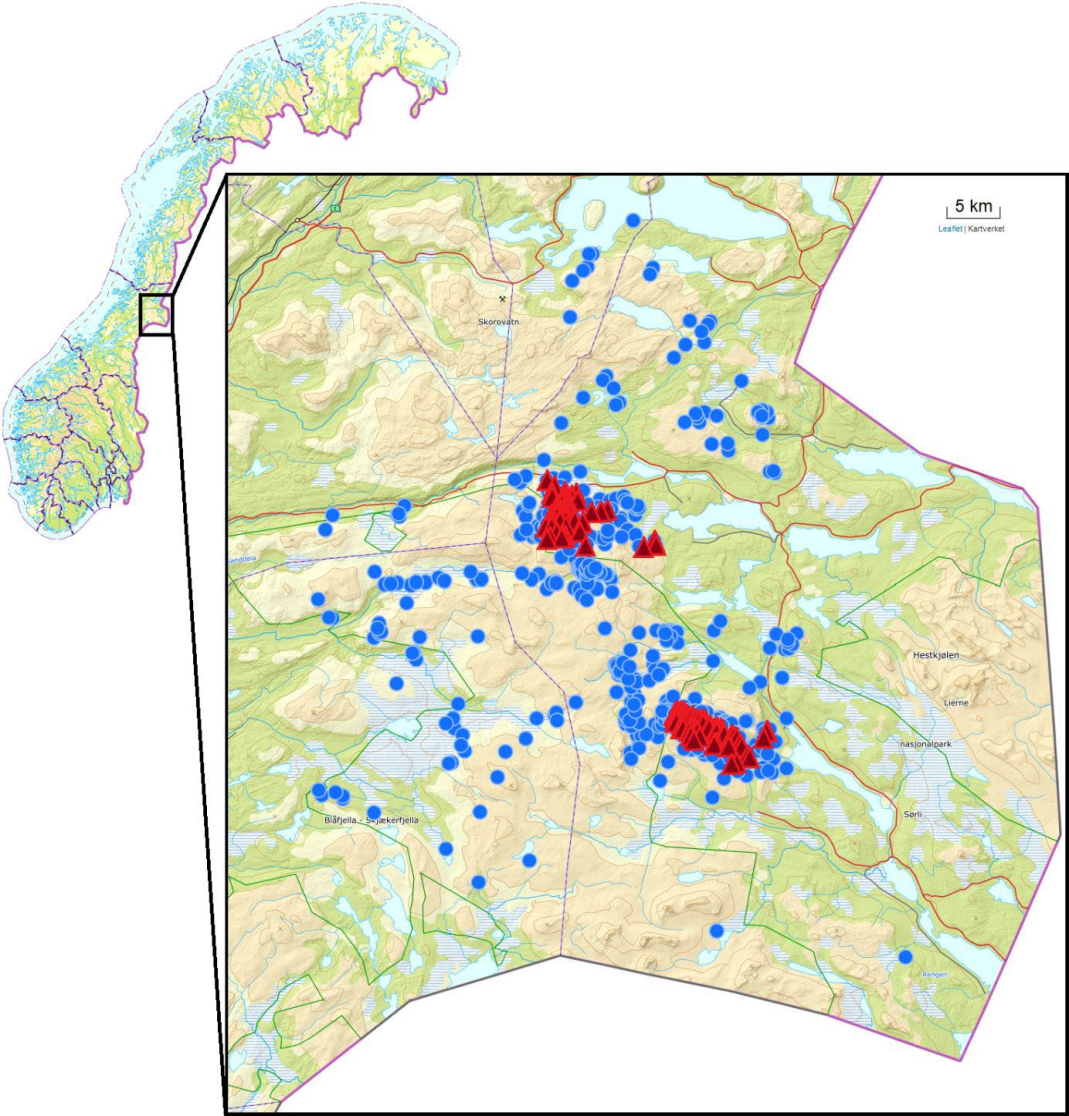
Study area (outlined box) showing all marking locations (red triangles) and telemetry positions (blue dots) of the marked birds. The northern cluster of red triangles represents the Lifjellet location and the southern cluster Gusvatnet

The basis of our field study was two locations, Lifjellet (64°25′–64°30′N, 13°11′–13°24′E, approx. 96 km^2^) and Gusvatnet (64°15′–64°18′N, 13°25′–13°37′E, approx. 54 km^2^), respectively, in Lierne Municipality in Central Norway, where all captures and marking of birds occurred (Figure 1). Because some birds migrated relatively long distances (>25 km, Arnekleiv, 2020), our dataset also includes several relocations in neighboring municipalities. Radio‐tagged willow ptarmigan were triangulated inside the total study area, as the birds dispersed or migrated out of the main areas. The capture sites for willow ptarmigan (see next section) spanned elevations from 456 to 759 m and were located in the subalpine to alpine bioclimatic zone. The subalpine bioclimatic zone was dominated by spruce (*Picea abies* L.) interspersed with birch (*Betula pubescens*). Dwarf birch (*Betula nana* L.) and willows (*Salix* spp.) comprise most of the shrubbery scattered among forest patches. At lower elevations, bogs/marshes are covered by grasses and sedges and the forests by ericaceous plants, while the vegetation at higher altitudes is dominated by dwarf birch heather, sedges, and lichens. The ground is typically snow‐covered from October until May. Main predators on adult willow ptarmigan observed in the study area include gyrfalcons (*Falco rusticolus* L.), golden eagles (*Aquila chrysaetos* L.), and red fox (*Vulpes vulpes* L.). Red fox and golden eagles predate on both nests (E. B. Nilsen, unpublished data) and adult willow ptarmigan (Munkebye, Pedersen, Steen, & Brøseth, 2003; Nyström, Ekenstedt, et al., 2006), while gyrfalcons mostly prey upon yearling or adult willow ptarmigan (Barichello & Mossop, 2011; Booms & Fuller, 2003; Nyström, Dalén, et al., 2006). In addition, arctic fox (*Vulpes lagopus* L.) and lynx (*Lynx lynx* L.) are present in the study area, but probably does not represent major mortality risks for ptarmigan in the study area due to low densities.

### 2.2 Field methods

During February and March 2015–2019, we captured a total of 188 willow ptarmigan at night using snowmobiles and large hand nets with prolonged handles, as described in Nilsen, Moa, Brøseth, Pedersen, and Hagen (2020). To prevent birds from flying off before the field personnel were close enough to capture them, a high‐powered headlamp was used to dazzle the birds. After capture, we placed the birds in an opaque bag to reduce stress. We aged the birds based on descriptions in Bergerud, Peters, and McGrath (1963) and Myrberget (1975), by examining the pigmentation on the outer primaries and categorized them as either juvenile (<1 year old) or adult (>1 year old). We assessed the sex of each bird in the field by visual inspection of morphological characteristics and later confirmed the sex by DNA analyses using a feather sample collected during capture. For 17 birds, we did not obtain any biological samples or the DNA analysis was unsuccessful and could thus not confirm sex using DNA. Based on the birds where both field‐based and DNA‐based sex determination were obtained (*n* = 166), field‐based determination was correct in 85% (141/166) of the cases. We therefore opted to include birds where sex was not verified using DNA analyses; we are aware that this induces a potential bias. Before releasing the birds, they were fitted with a uniquely numbered leg ring (~2.4 g) and a Holohil RI‐2BM or Holohil RI‐2DM radio transmitter (~14.1 g). The radio transmitters had an expected battery lifetime of 24 months (RI‐2BM) or 30 months (RI‐2DM) and included a mortality circuit that was activated if a bird had been immobile for 12 hr. For all marked birds, the combined weight of the leg ring and radio transmitter was <3.5% of the body mass. From the total number of birds that we instrumented with VHF radio collars (*n* = 188), some birds (*n* = 6) were never relocated after release and were thus excluded from the study. This left us with a total sample of *n* = 182 individual willow ptarmigan included in the analyses. Of these birds, there were 53% females and 47% males. During the study period, we recorded mortalities for 124 birds (i.e., 68% of all birds marked), whereas 58 birds (32%) were censored either because we lost contact or because they were alive at the end of the study period (Table 1). As previous studies did not find adverse effects of radio tags on survival (Hannon et al., 2003; Terhune, Sisson, Grand, & Stribling, 2007; Thirgood, Redpath, Hudson, Hurley, & Aebischer, 1995), we assumed the radio tags would not influence the survival of willow ptarmigan.

**TABLE 1:** Number of radio‐tagged birds and mortalities for each calendar year of the study

Year	2015	2016	2017	2018	2019	Total	Total in analysis	Prop. Mort.	Prop. Surv.
Tagged birds	32	38	40	38	40	188	182	(124/182)	(58/182)
Mortalities	19	21	34	30	20	124	124	0.68	0.32

Note

Also shown are the total number of birds used in the analysis and the number of these that died or survived until the end of the study.

Following release of the radio‐tagged birds, they were triangulated from the ground at least once a month for 10 months of the year (February–November) by qualified field personnel. If a mortality signal was heard from the transmitter, we recovered it as soon as possible to determine cause of death. A number of birds dispersed out of the main study areas and were thus out of signal range for field personnel on the ground. To avoid loss of data, we conducted aerial triangulation using a helicopter or airplane three times a year (May, September, and November) in the years 2016–2019. In 2015, we only conducted one triangulation from the air in October.

The data used here are based on an ongoing field project, and the dataset is therefore continuously updated as new data are registered. For analyses reported here, we used data collected between 16.02.2015 and 27.11.2019. Data used in this article are archived and openly available at Israelsen, Eriksen, Moa, Hagen, and Nilsen (2020).

### 2.3 Individual capture histories

As a basis for our analysis of annual survival probabilities, we set 1 August to represent the start of the biological year. This choice made it possible to directly compare our results with those from previous studies in Scandinavia (Sandercock et al., 2011; Smith & Willebrand, 1999). With the redefined year, the first time period of the study started on 1 August 2014 and ended on 31 July 2015, while the final time period (6 in total) started on 1 August 2019 and ended on 31 July 2020. Hereafter, “year” refers to the biological year from 1 August to 31 July.

In addition to the analysis of annual survival probabilities, we also assessed patterns of survival in four distinct seasons. First, we defined the autumn season as 1 September to 30 November. This season is strongly affected by the annual recreational harvest season starting 10 September, and previous studies from Scandinavia have shown that harvest is a dominating mortality factor in autumn (Sandercock et al., 2011; Smith & Willebrand, 1999). Most of the hunting effort usually takes place during the first weeks after the hunting season has started (Smith & Willebrand, 1999; Willebrand, Hörnell‐Willebrand, & Asmyhr, 2011). In our case, there were only two harvest‐related mortalities outside the defined autumn season (during the winter harvest season in February); these were included as mortalities in the winter and full‐year analyses. Second, we defined the winter season as 1 December to 31 March. Winter survival of willow ptarmigan in Scandinavia has typically been found to be high (Sandercock et al., 2011; Smith & Willebrand, 1999). Finally, we defined the mating and prebrooding period as the spring season (1 April to 31 May), while the incubation and chick‐rearing period were defined as the summer season (1 June to 31 August). The age of each bird (juvenile vs. adult) was estimated at capture in February/March and separated into two age categories (<1 year old and >1 year old). In the further analyses, age was only included as a predictor variable for the spring survival analysis, as the presence of juvenile willow ptarmigan could only be known with certainty for the spring season. Given that juveniles were approx. 9 months old at capture, there were no marked juveniles present during the autumn season for our study. Further, a comparison between the survival of “yearlings” and adults could not be made due to the low number of yearlings still alive in the autumn and winter months.

Based on the time schedules described above, we constructed capture histories for each bird following a time‐to‐event modeling approach (Pollock, Winterstein, Bunck, & Curtis, 1989). Birds that were alive at the end of the year (31 July) or season (see above for definitions) were censored and re‐entered in a new row in the dataset for the next year or season. All juvenile birds alive at the end of the year were re‐entered as adults at the start of the new year (1 August). Thus, each observation in the dataset is one bird in one given year. For all years in total, we had 350 observations or “bird‐years.” Naturally, with only one tagging session in February/March the number of observations available for analyses decreased due to mortalities from winter (*n* = 251), spring (*n* = 232), summer (*n* = 206) to autumn (*n* = 161). In addition to the capture‐related variables (ring identification number, sex, and age), five new variables were created: time period, entry day, exit day, event (if the bird was alive = 0 or dead = 1), and cause of death (harvest = 1 or natural = 2). Natural causes were defined as any non‐harvest‐related mortality. All unknown mortality causes were assumed to be natural (since harvested marked ptarmigans were reported), but not identifiable to a single natural cause. We assumed that all harvested birds were reported as harvested. Hunters were frequently reminded to report and return radio tags and/or leg rings, and since marked birds were not banned from harvest, this should be a valid assumption. Nevertheless, some harvested birds may not have been reported and could thus yield a slight underestimation of harvest mortality rates.

Because the birds were not monitored in continuous time, the exit date (i.e., date for mortality or censoring) had to be estimated in many cases. For birds that were alive at the end of the study, exit day was set to the day that they were last confirmed to be alive. Birds that died due to natural causes had their exit day defined as the midpoint between the last day they were heard alive and the first time the mortality signal from the transmitter was heard. For birds that were shot by hunters, exit day was set according to the day the bird was shot, as reported by the hunters. A few birds (*n* = 4) that were censored due to loss of contact (radio transmitter failure or other) re‐entered the study when they were reported as shot, and their status was changed to alive until the day they were shot.

### 2.4 Survival analyses

Survival rates were estimated using 5 years of radio telemetry data, collected between 2015 and 2019 in Lierne, Snåsa, Grong, and Røyrvik municipalities. We applied Pollock et al. (1989) staggered‐entry modification of the Kaplan–Meier procedure (Kaplan & Meier, 1958) to estimate annual and seasonal survival rates on a daily basis in the statistical software R, version 3.6.1 (R Core Team, 2019), employing functions from the *survival* package (Therneau, 2015). Other analyses and data handling were also conducted in R.

To examine variation in mortality risk due to sex, age, and year, we used Cox proportional hazards regression models fitted using the function *coxph* (Therneau, 2015). To account for nonindependence caused by the fact that some individuals were represented by more than one observation, individual ID (ring identification number) was included as a random variable. The proportional hazards assumption of all Cox regression models was assessed by running model diagnostics with the *cox.zph* function (Therneau, 2015). Annual cause‐specific mortality under the competing risks of natural and harvest mortality was estimated by employing a nonparametric cumulative incidence function estimator (NPCIFE) described by Heisey and Patterson (2006), using the code modified by Sandercock et al. (2011). The same procedure was also used to estimate the cumulative risk of natural and harvest mortality during autumn only. To test for any dependencies in harvest or natural mortality risk due to sex, we used a stratified Cox proportional hazards analysis. We first stratified the data by mortality cause (natural or harvest) and then ran two separate Cox proportional hazards regressions, one for natural mortality risk and one for harvest mortality risk, testing for an effect of sex in each model. Finally, we estimated separate continuous annual hazard functions for both mortality causes combined, for harvest mortality only and natural mortality only by employing Gu (2014) smoothing spline functions.

All survival analysis figures were created using package *ggplot2* (Wickham, 2016), while the map in Figure 1 was created with packages *leaflet* and *mapview* (Appelhans, Detsch, Reudenbach, & Woellauer, 2020; Cheng, Karambelkar, & Xie, 2019).

All codes used to perform analysis and produce graphics presented in this article are archived and openly available as an registration (time‐stamped and published archive) at Israelsen et al. (2020).

## 3 RESULTS

### 3.1 Annual survival probabilities

**FIGURE 2:**
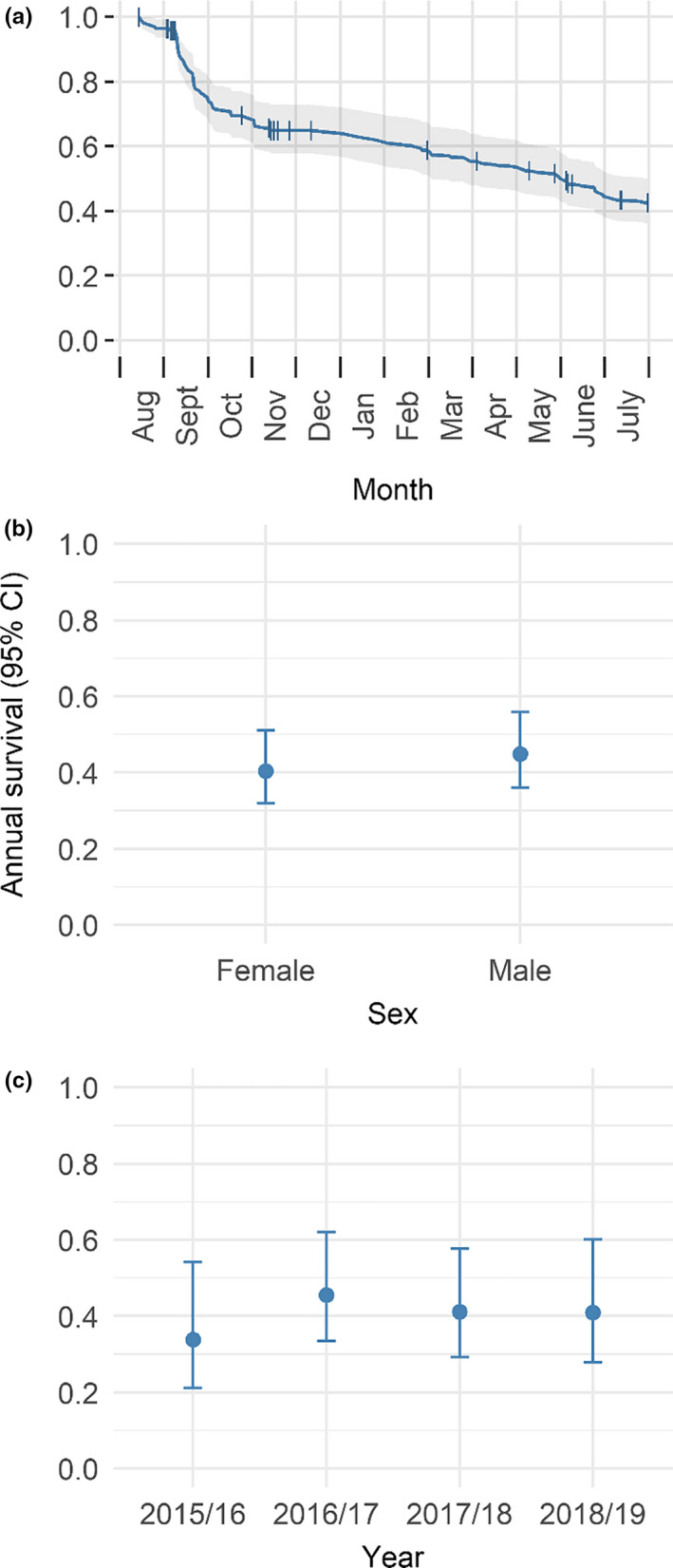
(a) Survival of willow ptarmigan 1 August–31 July (vertical lines represents censoring events). (b) Annual survival for each sex and (c) annual survival for complete willow ptarmigan years

Annual survival probability of willow ptarmigan across all years was estimated to be 0.43 ± 0.03 SE (Figure 2a). For females and males, annual survival was estimated to be 0.40 ± 0.05 SE and 0.45 ± 0.05 SE, respectively (Figure 2b). When stratified by sex, the proportional hazards assumption was not met (*χ* ^2^ = 5.71, *p* = .02), and we therefore did not use Cox proportional regression to assess this difference statistically. We further examined if there was any between‐year variation in annual survival (Figure 2c), but no significant between‐year variation in annual survival was found (Wald test = 1.67, *df* = 3, *p* = .60). The assumption about proportional hazards for the global model was met (*χ* ^2^ = 7.27, *p* = .06). Therefore, annual survival estimates remained relatively stable for all years.

### 3.2 Seasonal survival rates

**FIGURE 3:**
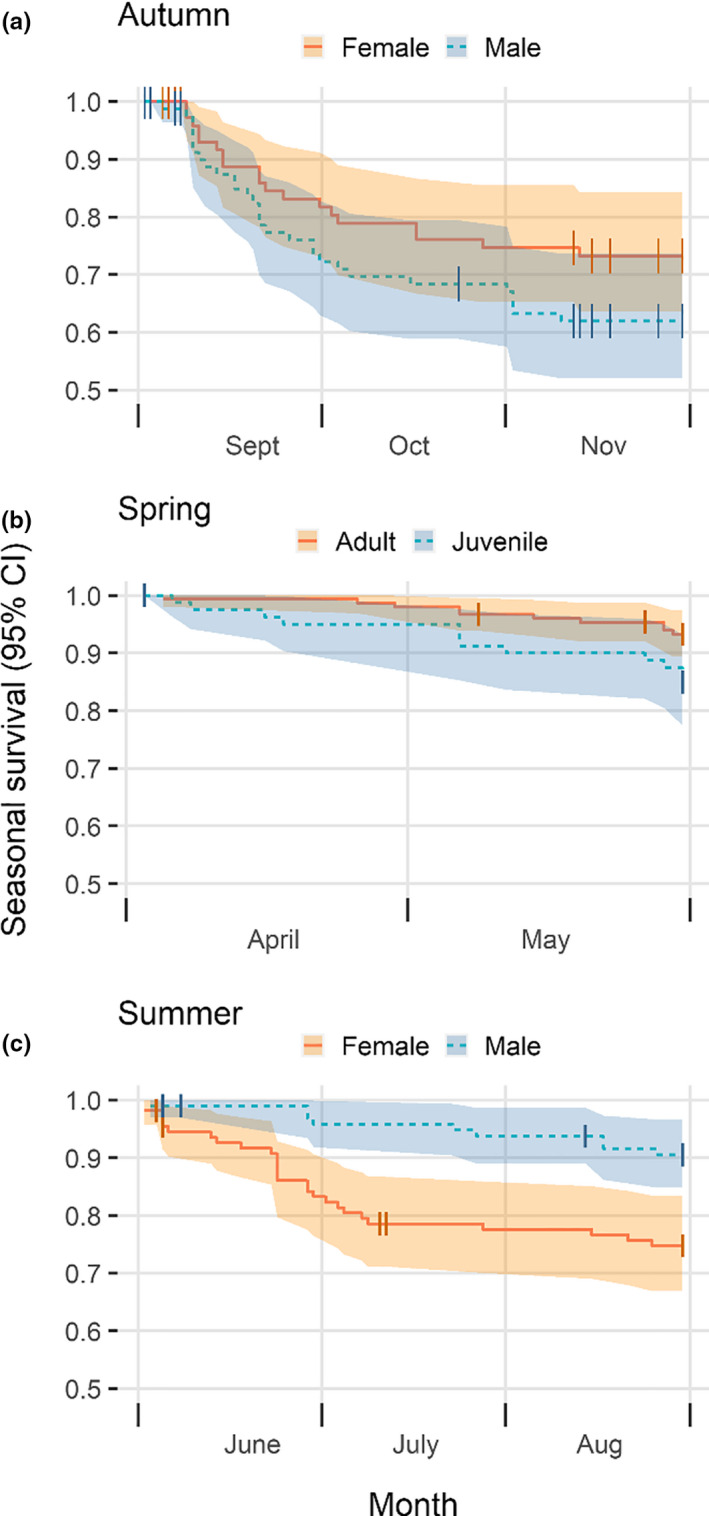
Seasonal survival in (a) autumn for males and females, (b) spring for juvenile and adults, and (c) summer for males and females. Vertical lines represent censoring events. Note that the probabilities on the y‐axis range from 0.5 to 1

In the second part of the analysis, we created distinct datasets for the various seasons (as defined in the methods) and estimated survival probabilities for each season separately. As expected, autumn survival was low (0.67 ± 0.04 SE), and there were some indications that males had higher mortality risk than females during this season (HR = 1.53, 95% CI = 0.90–2.60, *z* = 1.58, *p* = .11; Figure 3a). The assumption of proportional hazards was met when stratified by sex (*χ* ^2^ = <0.01, *p* = .98). During the winter season, overall survival probability was high (0.90 ± 0.03 SE), with no discernible difference in mortality risk between males and females (HR = 0.65, 95% CI = 0.24–1.78, *z* = −0.84, *p* = .40). The assumption of proportional hazards when stratified by sex was met (*χ* ^2^ = 2.28, *p* = .13). Also during spring, survival probabilities were high (0.90 ± 0.02 SE). The proportional hazards assumption was met for sex (*χ* ^2^ = 0.07, *p* = .79) and age (*χ* ^2^ = 0.08, *p* = .78), for the spring survival data. There was no difference in survival between males and females (HR = 1.10, 95% CI = 0.47–2.58, *z* = 0.23, *p* = .82) in spring, but juveniles (<1 year old) had a substantially higher risk of mortality than adult birds (HR = 2.35, 95% CI = 1.01–5.45, *z* = 1.98, *p* = .05; Figure 3b). During the three‐month‐long summer season, survival probability was lower than both winter and spring survival (0.82 ± 0.03 SE), and males had a substantially lower mortality risk than females (HR = 0.33, 95% CI = 0.16–0.69, *z* = −2.93, *p* = <.01; Figure 3c). The summer survival data for sex met the assumption of proportional hazards (*χ* ^2^ = 3.09, *p* = .08). For all seasonal analyses, year did not explain a significant amount of the variation in mortality risk for any season and the proportional hazards assumption was met for all seasonal data (except summer) used to test for effects of year (see Appendix).

### 3.3 Temporal variation in cause‐specific mortality risk

**FIGURE 4:**
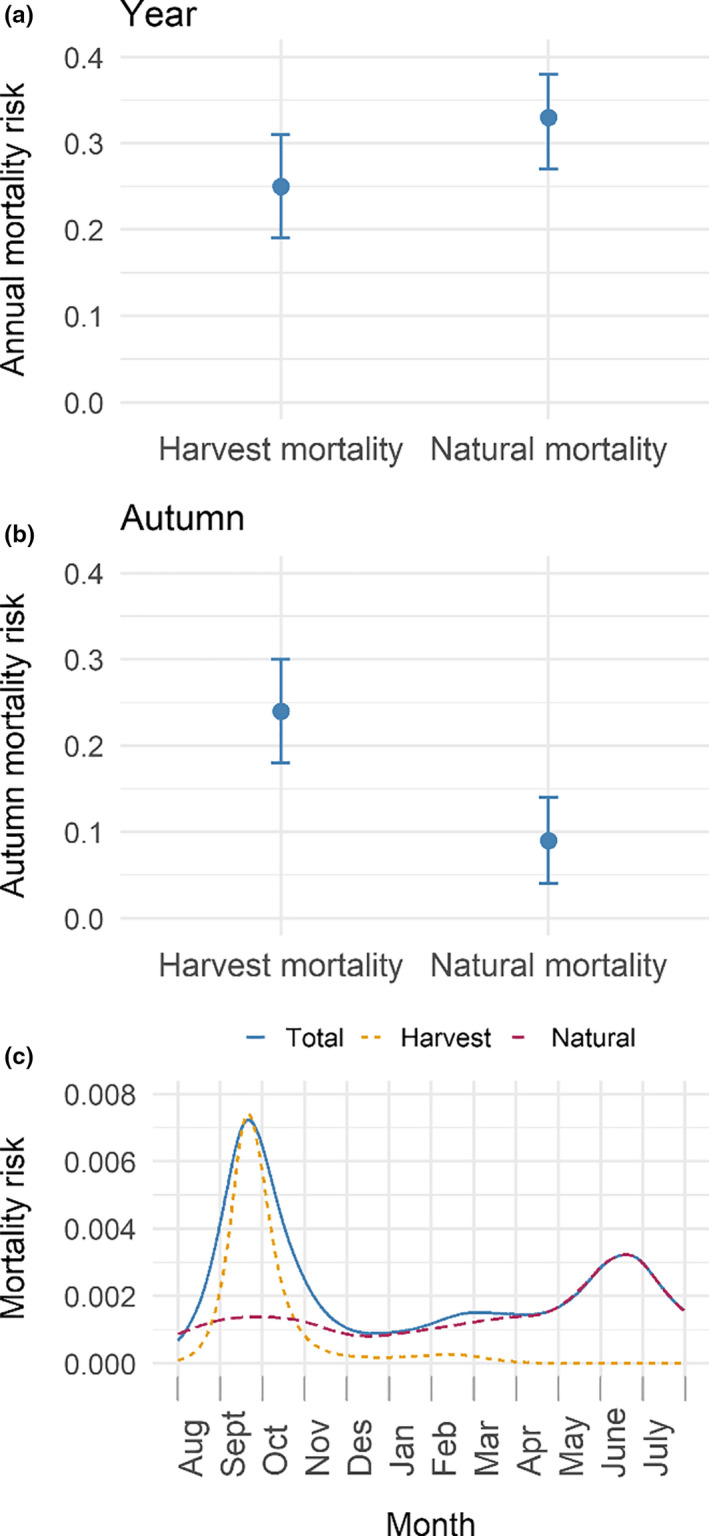
(a) Annual mortality probability due to natural causes and harvest. (b) Autumn mortality probability due to natural causes and harvest. Note that the range of probabilities on the y‐axis goes from 0 to 0.40 for (a) and (b). (c) Smoothed instantaneous hazard function showing daily hazard risk for total, harvest, and natural mortality

In the third and final part of the analyses, we investigated annual and seasonal cause‐specific mortality risk. Annually, there was a higher probability of mortality due to natural causes (CIF = 0.33 ± 0.03 SE, 95% CI = 0.28–0.38) than being shot (CIF = 0.25 ± 0.04 SE, 95% CI = 0.19–0.31) for willow ptarmigan in this study (Figure 4a). Unsurprisingly, this relationship was reversed when we examined the autumn season only, with harvest mortality being substantially higher (CIF = 0.24 ± 0.04 SE, 95% CI = 0.18–0.30) than the probability of dying of natural causes (CIF = 0.09 ± 0.03 SE, 95% CI = 0.04–0.14; Figure 4b). We did not find any clear difference in mortality risk between males and females for the risk of being shot (HR = 1.51, 95% CI = 0.81–2.81, *z* = 1.28, *p* = .20) or dying of natural causes (HR = 1.60, 95% CI = 0.53–4.82, *z* = 0.83, *p* = .41).

Finally, we estimated smoothed instantaneous mortality risk for natural and harvest mortalities combined (total), harvest mortalities only, and natural mortality only (Figure 4c). In general, the mortality risk was highest in September and October, coinciding with the first few weeks of the hunting season (10 September to 28 February). During winter and early spring, mortality risk was very low, but increased slowly and gradually until mid‐June, yielding another peak in mortality risk. The risk of harvest mortality is mainly relevant in the autumn, and the spring peak in mortality risk is driven exclusively by natural mortality factors.

## 4 DISCUSSION

### 4.1 Survival

**FIGURE 5:**
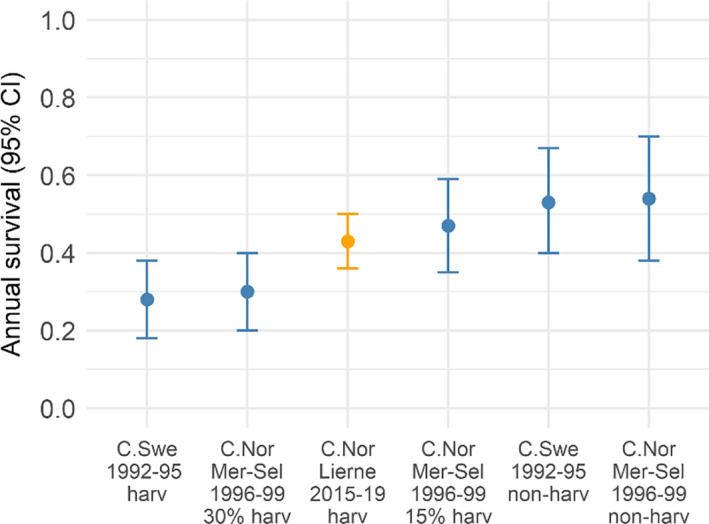
Annual survival estimates for this study (Lierne 2015–2019, harvested area, in orange) in comparison with what was found in Sandercock et al. (2011; Meråker‐Selbu in central Norway, nonharvested area, 15% and 30% harvest rate) and Smith and Willebrand (1999; central Sweden harvested area and central Sweden nonharvested area)

In our study area, we estimated annual survival to be 0.43 ± 0.04 SE, with no discernible distinction between years. This annual survival probability is comparable to previous studies in other localities in Norway (Figure 5; Sandercock et al., 2011) and North America (Martin, Hannon, & Rockwell, 1989; Sandercock, Martin, & Hannon, 2005). Annual survival in our study area was lower than the estimates by Sandercock et al. (2011) for annual survival in nonharvested areas (0.54, 95% CI = 0.38–0.70) and areas with experimental treatments of 15% harvest (0.47, 95% CI = 0.35–0.59), as well as the estimate in Smith and Willebrand (1999) for nonharvested areas (0.53, 95% CI = 0.40–0.67). However, the survival probability found in our study area was higher than that reported under 30% experimental harvest in central Norway (Sandercock et al., 2011; 0.30, 95% CI = 0.20–0.40) and under harvest in central Sweden (Smith & Willebrand, 1999; 0.28, 95% CI = 0.18–0.38, Figure 5). During our study period (2015/16 to 2019/20), local management reported an average harvest rate of 18% (T. Åberg, personal communication, June 25, 2020), based on estimated population size and total bag size, in our study region in Lierne Municipality. Taken together, these studies indicate that higher harvest rates yield lower annual survival of willow ptarmigan, which further demonstrate that harvest mortalities are at least partially additive to natural mortalities. This gives some insight into the importance of harvest intensity on annual survival for willow ptarmigan in Scandinavia. We did not find any clear difference in annual survival between males and females. This might be the result of counteracting seasonal effects, as suggested by Hannon et al. (2003); in general, we found that males tended toward lower survival (although not statistically significant) than females in autumn, while females had substantially lower survival than males during summer.

In winter and spring, survival was generally high, and there were no clear signs of sex differences in survival. However, juvenile birds had much lower survival in spring than adult birds. Willow ptarmigan vigorously defend their established territories from any intruders, including juveniles (Eason & Hannon, 2003; Pedersen, Steen, & Andersen, 1983; Rørvik, Pedersen, & Steen, 1998). We expect that inexperienced yearlings trying to acquire a territory may be less alert to predators during this time and may therefore suffer greater mortality risk than adults. This difference might arise due to differential predation pressure, and Barichello and Mossop (2011) suggested that gyrfalcon exerts higher predation pressure on young ptarmigan compared to adults. Such a preference would indicate that juveniles are easier prey than adult birds and could explain the lower survival of juveniles in spring found in this study. Inexperience may also affect the foraging ability of young birds during winter–spring, resulting in poor spring body condition (Wiebe & Martin, 1998).

We also found a distinct difference in survival between males and females during summer, with female willow ptarmigan having markedly lower survival compared to males. Hannon et al. (2003) suggest that female willow ptarmigan are more prone to predation in the breeding season than males as a result of their great parental investment. This investment includes the process of egg laying and incubation, as well as any clutch defense behavior toward predators (Martin & Horn, 1993). Both male and female willow ptarmigan defend the nest from predators, although males for the most part indirectly defend the nest by defending their female partner (Martin, 1984; Martin & Horn, 1993). The higher survival of males during summer suggests that they do not invest as much in the nest and are therefore in better condition than females during this time, allowing them to more effectively avoid predation.

There was no significant distinction in autumn survival between male and female willow ptarmigan, but our results did provide some indications that females have higher survival during autumn. Because our sample size in autumn is lower than in the other seasons resulting from mortalities between winter tagging and autumn, the power to detect any trend is also lower in autumn compared to the other seasons.

### 4.2 Cause‐specific mortality risk

In our study, we found that natural mortality risk varied throughout the year, revealing a minor peak in late September and a major peak in mid‐June. Sandercock et al. (2011) found a very similar pattern, although they reported an autumn peak that was more distinct and a summer peak that occurred somewhat earlier than mid‐May. In our study, the summer peak in natural mortality risk (Figure 4c) coincided with late incubation or hatching stage, a period which has previously been associated with high mortality risk (Winder et al., 2014, 2016). The reason for this heterogeneity between the studies is yet unknown. Differences in climate between the two locations could explain the observed distinctions, with the Lierne study area being located both further north and further inland than Meråker‐Selbu, which may cause the breeding dates of willow ptarmigan and/or predators to differ between the two areas. The distance and distinct climates between Lierne and Meråker‐Selbu mean that there could also be spatial differences in the predator communities of the two areas as well, yielding differing mortality risk patterns. Moreover, our data were collected approximately 20 years later than the data analyzed by Sandercock et al. (2011), which means that temporal changes to the predator community are also a potential explanation for the observed differences.

As expected, the vast majority of harvest mortalities occurred during the first weeks of the autumn hunting season, and the annual patterns in harvest mortality risk were mostly driven by these weeks. We found our estimated harvest mortality risk (0.24 ± 0.04 SE) to be identical to the estimate of hunting mortality in autumn in central Sweden (Smith & Willebrand, 1999). It is important to note that the core areas in our study (Gusvatnet and Lifjellet) are easily accessible, and areas close to infrastructure are often associated with higher hunting effort compared to more remote locations (Breisjøberget et al., 2018; Brøseth & Pedersen, 2000).

We found no significant autumnal difference between the sexes for either natural mortality risk or harvest mortality risk. Asmyhr et al. (2012) were also unable to find an effect of sex on harvest risk in a harvested area in central Sweden. Interestingly, Sandercock et al. (2011) showed that females were more at risk of harvest mortality under experimental harvest. In their experiment hunters mostly used pointing dogs during the hunt (Sandercock et al., 2011), while our study area had a mix of hunters with and without dogs (N. V. Bratlandsmo, personal communication, April 8, 2020). Male and female willow ptarmigan are to different degrees following the brood during the autumn hunting season, and this may affect the susceptibility for being shot (Bunnefeld, Baines, Newborn, & Milner‐Gulland, 2009). We speculate whether this grouping behavior may have different effects on harvest with or without dogs. As using a hunting dog usually gives the hunter more time to prepare before firing in each situation, it is likely that hunters may have time to shoot more individuals from large coveys of ptarmigan than if hunting without a dog. Since females are more prone to grouping, this might imply that more females may be shot when hunting with dogs than without, which would give a possible reason for the observed differences between our study and Sandercock et al. (2011).

### 4.3 Harvest management

In our study area, the willow ptarmigan harvest mortality risk was substantially higher than what is generally considered to be compensatory (Sandercock et al., 2011). Moreover, there seems to be a clear connection between harvest rate and willow ptarmigan survival, where willow ptarmigan in nonharvested areas have higher survival (Figure 5). It is therefore important to implement harvest strategies that can reduce risks of overharvest. Threshold harvest strategies have often been proposed as a way to counterbalance risk of harvest, especially when the exploited population occurs at low densities (Eriksen, Moa, & Nilsen, 2018), as it only permits harvest above a certain population threshold (Lande, Sæther, & Engen, 1997). Although, it does imply no harvest in the years where the population size is below this threshold (Lande et al., 1997).

## 5 CONCLUSION

The temporal resolution of this study allowed us to accurately estimate willow ptarmigan annual and seasonal survival, as well as cause‐specific mortality risks. We concluded that seasonal patterns in mortality might differ between demographic groups and that these differences might not be visible when analyzed at a coarser temporal resolution. Such patterns might be important when seeking to understand the evolution of life histories in fluctuating environments. Our results also provide insights into the relative importance of harvest and natural mortality for overall survival probability. Although natural mortality risk outweighed the estimated harvest mortality risk on an annual basis, harvest still constituted a relatively large percentage of mortalities observed. Comparison with nonhunted populations supports the view that such harvest mortality is at least partially additive. By identifying demographic differences in mortality risk throughout the year, our results are applicable for highlighting areas where conservationists or small game area managers should focus their efforts.

## CONFLICT OF INTEREST

None declared.

## AUTHOR CONTRIBUTIONS

**Markus F. Israelsen:** Conceptualization (equal); formal analysis (lead); writing – original draft (lead); writing – review and editing (equal). **Lasse F. Eriksen:** Conceptualization (supporting); data curation (supporting); investigation (supporting); supervision (supporting); writing – original draft (supporting); writing – review and editing (supporting). **Pål F. Moa:** Conceptualization (supporting); funding acquisition (supporting); project administration (supporting); supervision (supporting); writing – original draft (supporting); writing – review and editing (supporting). **Bjørn R. Hagen:** Conceptualization (supporting); investigation (supporting); writing – review and editing (supporting). **Erlend B. Nilsen:** Conceptualization (lead); data curation (lead); formal analysis (supporting); funding acquisition (lead); methodology (lead); project administration (lead); supervision (lead); writing – original draft (supporting); writing – review and editing (equal).

## Data Availability

Data and code used in this article are archived and openly available as an registration (time‐stamped and published archive) at Isralsen et al. (2020).

## APPENDIX 1

### Between‐year variation in seasonal survival

We did not find any significant between‐year variation in seasonal survival (Table A1).

**TABLE A1:** Test of between‐year variation in survival for each season. Shown here is the Wald test score, the degrees of freedom (*df*) and the *p*‐value (*p*)

Season	Wald test	*df*	*p*
Autumn	2.71	4	0.60
Winter	1.58	3	0.70
Spring	3.23	3	0.40
Summer	1.86	3	0.60

The assumption about proportional hazards for the models testing between‐year variation was met (Table A2).

**TABLE A2:** Test for the assumption about proportional hazards showing the chi‐square value (*χ* ^2^), the degrees of freedom (*df*) and the *p*‐value (*p*)

Season	*χ* ^2^	*df*	*p*
Autumn	1.57	4	.81
Winter	3.92	3	.27
Spring	4.30	3	.23
Summer	8.16	3	.04
